# Meetings of Societies

**Published:** 1911-06

**Authors:** 


### MEETINGS OF SOCIETIES.

### Bristol Medico-Chirurgical Society.

*March 8th*, 1911.

Dr. Bertram M. H. Rogers, President, in the Chair.

Mr. E. W. Hey Groves read a paper on **Resection of Posterior Nerve Roots** for (1) pain, (2) visceral crises, and (3) spasm. The histories of cases of each type—one of each—were given, and the technique of the operation and the results which had been achieved were described. (See page 105.) The paper was discussed by Dr. Michell Clarke.

Dr. D. A. Alexander described a case of **Portal Pyæmia.** The patient had suffered for several years from attacks of abdominal pain resembling (right) renal colic. With such an attack coincided in February, 1910, a sloughing hæmorrhoid, the ostensible source of portal infection. Jaundice was an early symptom ; rigors, frequent and severe, continued for three weeks, with hyperpyrexia, ecchymoses, and subcuticular abscesses. There was no hepatic enlargement. Crural thrombosis occurred, eventually with suppuration. In two months, with cardiac exhaustion, free fluid began to collect in the abdomen. The septic process ended with the evacuation of the crural abscess, and the ascites from portal obstruction was relieved by paracentesis. Recovery slowly ensued. No organism could be found in the urine or blood at the beginning of the illness, and thus no autogenous vaccine could be prepared, and that obtained from the streptococcus brevis of the crural abscess came too late for its value to be proved.

Dr. Carey Coombs read a paper on the **Histology of the Rheumatic Infection.** The histological changes met with in the four cardinal manifestations of rheumatic infection—polyarthritis, carditis, chorea, and subcutaneous nodes—may be described under the headings " general " and " special." *General.*—The morbid processes at work are proliferation (of endothelium and connective tissue in the formation of capillaries and of certain special cells), exudation (of fibrin, serum and leucocytes, especially of mononuclear types), and degeneration. Three grades of concentration are recognisable : diffuse degeneration of special cells, moderately diffuse leucocytosis and connective-tissue proliferation and formation of " nodules," aggregations of large multinuclear cells on a background of fibrin. *Special.*—In the heart nodules appear deep to the surface in the muscular and both serous layers, which show the other changes also. Nodes beneath the skin are probably aggregations of nodules. In inflamed joints subendothelial nodules are also found. The brain shows widespread chromatolysis, but no inflammatory changes of any kind. Examination of other organs (kidney, thyroid, etc.) has discovered certain morbid changes, but no inflammatory reaction. Chronic streptococcal malignant endocarditis is unlike rheumatic carditis in histology, but one case of post-scarlatinal carditis proved to be rheumatic in type. Experimental rheumatic lesions are in several essentials closely similar to clinical rheumatic phenomena. The paper was discussed by Drs. SCOTT WILLIAMSON, J. O. SYMES, MICHELL CLARKE and EDGEWORTH.

---

*April 12th*, 1911.

Dr. BERTRAM M. H. ROGERS, President, in the Chair.

The President announced that he had been invited to join the Lord Mayor's Coronation Committee as the representative of the Society, this being the first occasion on which the Society had been recognised as a body of public importance.

A letter from the Registrar of the University of Bristol was read giving notice of determination of the agreement made in 1893 with the Society as from March 25th, 1912.

The President explained that the University authorities wished to move the Medical Library from the present room to new premises in the old Blind Asylum buildings, and to provide there another reading-room for the members of the Society in place of that now in use. Meetings would be held in the present room even after the removal of the books. He had no doubt that a new agreement acceptable by both parties would be arrived at. After a short discussion Mr. Munro Smith proposed and Dr. Carey Coombs seconded: "That the Society has full confidence in the Committee's management of this business, and leaves the necessary arrangements in the Committee's hands." This was carried unanimously.

Dr. EDGEWORTH read a paper on **A Family Type of General Œdema.** In infants there occurs a type of general œdema not associated with albuminuria or kidney disease: its origin is obscure, and its result usually fatal. He had recently seen four fatal cases in a single family, in which the disease manifested its presence at ages varying from a few months to three and a half years. The children were not affected simultaneously; six years elapsed between the death of the first and last child.—Dr. W. SWAYNE asked if there was any connection between this condition and the cases of congenital œdema, with ascites and placental disease in the fœtus.—Dr. Carey Coombs inquired whether the manner of death threw any light on the cause.—Dr. E. C. Williams said the condition was rare. He had seen a case with diarrhœa and tetany, probably an intestinal toxæmia.—Dr. Fortescue-Brickdale commented on the rarity of the family type. He asked if there was ever effusion into the serous sacs.—Dr. Nixon held that the condition was an urticaria, which explained the non-occurrence of serous effusions.—Dr. Shingleton Smith inquired as to the condition of the thyroid and thymus glands in Dr. Edgeworth's cases, and alluded to sclerema neonatorum as possibly allied.—Dr. Edgeworth replied.

Dr. J. O. Symes showed cases of (1) **Pneumothorax,** (2) **Lateral Sclerosis,** and read a paper entitled "**Some Observations on School Hygiene**" (*vide* p. 109).—Dr. Watson Williams questioned the practical value of isolation of infectious exanthemata in school children. He referred to the swimming bath as a vehicle of contagion.—Dr. Fortescue-Brickdale, commenting on the relative infrequency of tuberculosis among Public School boys, suggested that they were as a rule not sent to school until after the age at which the disease is manifest or suspected.—The President contrasted the morbidity of Public School boys with the inmates of industrial schools. He also spoke of the superior training of the Oxford crew over the Cambridge as shown in this year's boat race.—Dr. E. C. Williams responded that the defeat of Cambridge was due to a want of speed rather than lack of stamina.—Dr. Brasher acknowledged that isolation in epidemics was scarcely justified by results, but he had also had experience of the disastrous results of deliberate exposure by parents of their children to acute exanthemata.—Dr. Nixon wondered whether, after all the disadvantages exposed in the discussion, school education was worth while.—Dr. Symes replied.

Dr. Watson Williams read notes of a **Case of an Operation for Suppurative Otitis, with Abscesses of Temporo-sphenoidal and Frontal Lobes,** showing the brain with the abscess cavities.—Dr. Brasher discussed the case.

Dr. E. C. Williams read notes of a **Case of Congenital Ataxia,** showing the patient, a child aged 3½ years, to the meeting.

---

*May 10th,* 1911.

Dr. Bertram M. H. Rogers, President, in the Chair.

Mr. H. F. Mole read a paper on **Plication of the Capsule of the Shoulder Joint for Habitual Dislocation.** The history of the methods of treatment successively adopted for the treatment of habitual dislocation was briefly recounted. The speaker's case was a woman of 24. She was an epileptic, and for two years in these fits the joint had been frequently dislocated, and latterly on other occasions, even as often as three times in one week. He had exposed the capsule by a vertical incision in front between the deltoid and pectoralis major, and exposed the capsule by dividing the subscapularis muscle. No undue laxity of the capsule was detected. Three thick cat-gut sutures were used to pucker the capsule in front. The arm was fixed to the side for three weeks. The muscles were found much atrophied by the end of that time. There had been no recurrence of dislocation to the present time, five months after operation. In that time she had had two fits.—Mr. Hey Groves doubted if laxity of the capsule was the cause of the dislocations, and thought that the good results of the operation might be due to division of the subscapular and other muscles. It was remarkable how free the joint movements were in the case, and contrasted with the proclivity of this joint to be stiffened by traumatism.—Dr. E. H. Stack alluded to the ease with which stiff shoulder joints could be dislocated by forcible movements.—The President asked if the weakness and wasting of the muscles which were still evident might not account for the absence of dislocation.

Mr. Munro Smith read a paper on **The Prevalence of Local Neuritis and Herpes zoster.** (The paper will be published in a future issue of this *Journal.*) The speaker suggested that acute brachial neuritis was a result of inflammation of the posterior root ganglion and analogous to herpes zoster, and that the inflammation was toxic and due to serous organism.—Mr. James Taylor thought that there was a traumatic element in such cases as those described.—Dr. Alexander thought there were cases of neuritis of a similar ætiology to herpes zoster, but without the herpetic eruption.—Dr. J. A. Nixon brought forward evidence derived from the statistics of the Bristol Royal Infirmary to show that herpes zoster occurred in epidemics.—Dr. Michell Clarke pointed out that the pain of herpes zoster might be present for some time before the eruption appeared. He had not found neuritis more common in his practice latterly than formerly. He thought that a posterior root ganglion affection would not explain the motor effects seen in the neuritis cases.—Mr. Munro Smith in reply said that in his cases there were no motor nerve effects beyond a little wasting of muscles from disuse.

Dr. A. Rendle Short read a paper (to be published later in this *Journal*) on **End Results of Operations in the Stomach.**

He surveyed the end results of 164 operations upon the stomach performed at the Bristol Royal Infirmary during the last nine years.—Mr. GROVES spoke of the great value of the statistics which had been collected. In gastro-enterostomy for pelvic lesions the benefit was much less if food continued to pass through the pylorus in considerable quantity, stenosis being absent.

Dr. MICHELL CLARKE showed a case of **Word Blindness.** The patient could write, but could not after a short interval read what he had written. Speech was perfect. There was alexia, but not agraphia. The possible situation of the lesion was explained with the aid of a diagram. The cause was probably a localised softening. The onset of symptoms had been sudden eighteen months previously.

Dr. MICHELL CLARKE also read notes on a **Case of Tumour of the Corpus Callosum**, and showed numerous macroscopical sections of the affected brain.

J. LACY FIRTH.

J. A. NIXON, *Hon. Sec.*

